# The GCC repeat length in the 5'UTR of *MRP1 *gene is polymorphic: a functional characterization of its relevance for cystic fibrosis

**DOI:** 10.1186/1471-2350-7-7

**Published:** 2006-02-07

**Authors:** Elena Nicolis, Matteo Pasetto, Cristina Cigana, Ugo Pradal, Baroukh M Assael, Paola Melotti

**Affiliations:** 1 Cystic Fibrosis Center, Azienda Ospedaliera di Verona, Piazzale Stefani 1, 37126 Verona, Italy

## Abstract

**Background:**

Among the members of the ATP binding cassette transporter superfamily, MRPs share the closest homology with the CFTR protein, which is defective in CF disease. *MRP1 *has been proposed as a potential modifier gene and/or as novel target for pharmacotherapy of CF to explain the clinical benefits observed in some CF patients treated with the macrolide AZM. The 5'UTR of the *MRP1 *gene contains a GCC triplet repeat that could represent a polymorphic site and affect the activity of the promoter.

**Methods:**

The *MRP1 *5' flanking region was amplified by PCR from 36 CF patients and 100 non-CF subjects and the number of GCC triplets of each allele was determined by sequence and electrophoretic analysis. We performed gene reporter studies in CF airway epithelial cells 16HBE14o-AS3, in basal conditions and in the presence of AZM.

**Results:**

We found that the GCC repeat is polymorphic, ranging from 7 to 14 triplets either in CF or in non-CF subjects. Our data are preliminary and have to be confirmed on a larger population of CF subjects. The transcriptional activity of the proximal *MRP1 *5' regulatory region revealed no statistically significant correlations between the number of repeats and treatment with AZM.

**Conclusion:**

We identified a novel polymorphism in the 5'UTR of *MRP1 *gene that provides multiple alleles in a gene relevant for multidrug resistance as well as for CF, determining that this region is transcriptionally active and that this activity does not appear to be influenced by AZM treatment.

## Background

CF is an autosomal recessive disease primarily manifested in the lung, which leads to respiratory failure. CF is caused by mutations of the *CFTR *gene whose product acts as a cAMP-activated chloride channel that is permeable to organic anions, including GSH [1,2]. A wide range of disease severities has been described, even among CF patients harbouring the same mutation. Therefore endogenous factors, that modulate or complement CFTR function, must exist. Because of the structural homology between CFTR and MRP1, a functional complementation of CFTR defect by MRP1 has been hypothesized [3]. Induction of MRP1 expression has been suggested to be responsible for improvement in lung function in a CF patient following chemotherapy with cyclophosphamide and epirubicin [4]. Low levels of MRP1 transcripts have been associated with more severe CF phenotype [5], while increased expression of the *MRP1 *gene has been associated with restored chloride conductance in a group of CF patients following treatment with AZM, giving rise to clinical beneficial effects [6,7]. *MRP1 *has been considered as a potential modifier gene [5] and/or as a target for therapy of CF in subjects responsive to AZM [8].

The *MRP1 *was originally cloned in a drug-selected lung cancer cell line [9]. MRPs are transmembrane proteins, whose mRNAs are detectable in most human tissues [10]. They share the closest homology with the CFTR protein, which belongs to the same C subfamily of the ABC transporter superfamily [11]. MRP1 confers resistance to chemotherapeutic drugs as well as to heavy metal oxyanions. It transports reduced glutathione conjugates, cysteinyl leukotriene LTC_4_, steroid glucoronides and bile salt derivatives in human cells, agents involved in the transcription factor NF-kB activation pathway, which has been reported to be altered in CF [12,13]. Therefore a functional link exists between genes involved in GSH metabolism and MRP1. NF-kB regulates expression of GSTP1 and polymorphisms of the anti-oxidizing enzymes GSTP1 and GSTM3 are associated with severity of CF [14]. The *GSTM1 *gene, encoding an enzyme that forms glutathione adducts, is deleted in most severe CF patients [15]. CF patients carrying a wild type allele for both GSTM1 and GSTT1 may be at reduced risk of severe lung disease. The abnormal reduced glutathione transport caused by CFTR mutation seems to play a critical role in the pathogenesis of CF and this parameter might also be complemented by MRP1 in CF patients. MRP1 has been also involved in the regulation of endogenous channels as chloride channels [16,17]. The physiological functions of MRP1 are the subject of many current investigations: the available evidence indicates that it is involved in detoxification, drug resistance, oxidative stress and inflammation [18,19]. An impaired response to inflammatory stimuli has been described in MRP1(-/-) knockout mice [20], even if *MRP1 *has not been demonstrated to be a disease-causing gene.

The sequence of the *MRP1 *gene has been reported [9]. The 5' flanking region is GC rich (88%) while the 5'UTR contains a GCC triplet repeat that has been hypothesized to represent a polymorphic site [12]. The influence of the number of triplets in the 5'UTR on MRP1 mRNA transcription, stability and translational efficiency has not been determined.

We searched a genetic marker for *MRP1 *with potential functional relevance aiming to establish: 1) whether the GCC repeat in the *MRP1 *5'UTR is polymorphic, 2) if the GCC triplets length could affect the activity of the promoter in CF airways epithelial cells either in basal conditions or upon AZM treatment.

## Methods

### Patients and controls

Genomic DNA from 100 unrelated non-CF subjects and 36 CF patients was collected. Nucleic acid was extracted from peripheral blood leukocytes using the salting out method [21]. Diagnosis of CF was based on clinical, biochemical and genetic data. The most common CF causing mutation, deltaF508, was detected in 24 patients, 7 of which were homozygous. This study was approved by the local institutional Ethic Committee.

### Genotyping

Specific primers were designed based on the nucleotide sequence of the 5' regulatory region of *MRP1 *gene (GenBank:U07050) (sequences are available upon request). PCRs were performed in an Applied Biosystems 9700 Thermal Cycler, using a final volume of 50 μl. Due to the high GC levels of the PCR products, 7-deaza-dGTP (Roche) was utilized instead of dGTP in the dNTPs mix. PCR-1 started with 350 ng of genomic DNA; 2.5 μl of PCR-1 were subjected to further amplification (PCR-2) and a third round of amplification (PCR-3) was performed with 2.5 μl of PCR-2. Amplifications were carried out starting with 10 min template denaturation/AmpliTaq Gold (Applied Biosystems) activation step at 95°C, followed by 40 cycles (20 cycles for PCR-3) of denaturation at 95°C for 30 s, annealing at 58°C for 30 s and extension at 72°C for 30 s. As a final product the fragment from -337 nt to -43 nt, starting from the translational starting site of the *MRP1 *5' gene, was obtained.

PCR products were purified with the NucleoSpin Extract kit (Macherey-Nagel). Samples were then sequenced using Thermo Sequenase II Dye Terminator Cycle Sequencing Premix Kit (Amersham Biosciences) and the automated 373A DNA Sequencer (Applied Biosystems).

PCR-3 was carried out in the presence of a primer conjugated to FAM dye (5-carboxyfluorescein) (Applied Biosystems) that generated labelled amplicons suitable for electrophoresis and Genescan analysis.

Genescan analysis Software (Applied Biosystems) was used to determine the length of the PCR products. In order to validate our method we utilized a vector including the *MRP1 *(GCC)7 allele (kind gift from Roger Deeley, Queen's University, Kingston, Ontario, Canada) as control. A proof-reading enzyme was utilized and at least duplicates of each sample and control vector were performed to exclude polymerase slippage.

### Gene reporter studies

The *MRP1 *5' regulatory region containing 7 or 14 GCC triplets, obtained as PCR-3 product, was cloned in the pGL3 vector (Promega). The constructs were checked by restriction, sequence and Genescan analysis and named MRP1(GCC)7-luc or MRP1(GCC)14-luc, according to the number of triplets. The CF airway epithelial cell line 16HBE14o-AS3 (kind gift from Pamela Davis, Case Western Reserve University School of Medicine, Cleveland, OH, USA) was transiently co-transfected using FUGENE (Roche) with the reporter vector MRP1(GCC)7-luc or MRP1(GCC)14-luc and a beta-gactosidase expressing vector, utilized for normalization. The day after the transfection, cells were incubated for 24 hrs with or without AZM 8 μg/ml (Pfizer Italia). This concentration is consistent with that achieved in the lung of CF patients treated with AZM [22]. Luciferase activity was determined using the Luciferase Assay System (Promega) according to the manufacturer's instructions. Luciferase activity in insert-less (pGL3) transfected cells was almost undetectable.

## Results

### The MRP1 5' flanking region is polymorphic

The *MRP1 *5' flanking region was amplified by PCR from 36 CF patients and 100 non-CF subjects and the number of triplets of each allele was determined by sequencing and electrophoretic analysis.

In non-CF subjects the number of triplets ranged from 7 triplets to 14. Six distinct alleles consisting of 7, 10, 11, 12, 13 and 14 GCC triplets were found. The frequencies of these *MRP1*(GCC)n alleles, where n indicates the number of triplets, is shown in Table 1. The genotypes frequencies of the GCC alleles are indicated in Table 2.

**Table 1:** *MRP1*(GCC)n alleles frequencies in non-CF and CF subjects

**Number of GCC triplets**	**Alleles in non-CF subjects (%)**	**Alleles in CF patients (%)**
7	32	29
10	3	0
11	16	21
12	13	10
13	31	38
14	5	2

**Table 2:** Genotypes frequencies of the *MRP1*(GCC)n alleles in the control group and in the CF patients

**Genotype**	**% in non-CF subjects**	**% in CF subjects**
7/7	23	25
13/13	15	6
7/13	12	0
11/12	10	11
11/13	9	0
12/13	7	28
7/11	5	6
12/12	4	3
11/11	3	0
14/14	3	0
10/11	2	8
10/13	2	0
10/14	2	0
13/14	2	2
7/14	1	0
12/14	0	8
7/12	0	3

### Assessment of transcriptional activity

**Figure 1:**
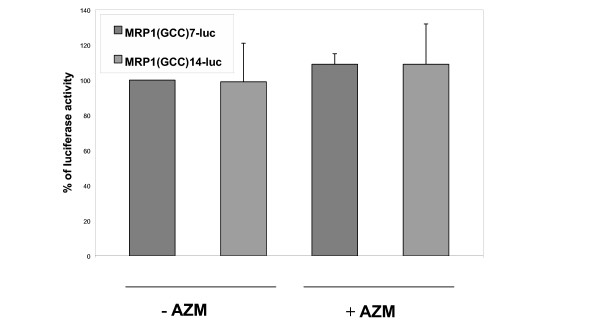
**Comparison of the transcriptional activity of MRP1 alleles containing 7 or 14 GCC triplets**. 16HBE14o-AS3 cells were transfected with reporter vectors including the *MRP1 *5'UTR region with 7 [MRP1(GCC)7-luc] or 14 triplets [(GCC)14-luc] driving the luciferase gene. 16HBE14o-AS3 cells transfected with MRP1(GCC)7-luc and not treated with AZM were considered as calibrator. Percentages of luciferase activity normalized with beta galactosidase are shown. Specificity of the signal was assessed by determining the luciferase activity in insert-less (pGL3) transfected cells, which was almost undetectable. Experiments were performed in triplicate (n = 3, p > 0.05 for all conditions, paired T-Test; SEMs are indicated by bars).

In order to establish whether the length of the GCC repeat can affect the transcriptional activity we performed gene reporter studies in CF airway epithelial cells 16HBE14o-AS3. This region has been previously demonstrated to be transcriptionally active by gene reporter studies [9]. Since the clinical status as well as the response to AZM has been associated to the levels of *MRP1 *mRNA in CF patients, we tested the transcriptional activity of *MRP1 *in our model by gene reporter assays in the presence or absence of the drug. The levels of luciferase activity measured in the constructs containing 7 or 14 GCC triplets of the *MRP1 *5' regulatory region were not significantly modified (p > 0.05) either in basal conditions or in the presence of AZM (Figure 1).

## Discussion

This study demonstrates that the genomic region from -337 nt to -43 nt of the *MRP1 *5' regulatory region includes a polymorphic site and possesses transcriptional activity. We also demonstrate that the *MRP1*(GCC)7 and *MRP1*(GCC)14 alleles (chosen as representative of the shortest and longest triplet length we identified) do not show any statistically significant differences in transcriptional activity when tested in CF airway epithelial cells *in vitro*. We chose these two alleles since we hypothesized that the length of the GCC repeat could influence *MRP1 *transcriptional activity as suggested by other authors [9]. Since we have been focusing to a partial region of the 5'UTR of the *MRP1 *gene, our approach was not aimed to test the influence of upstream regulatory sites eventually associated with specific GCC(n) alleles, as a recently described G/C single nucleotide polymorphism [23].

The number of alleles described in our limited group of CF subjects does not allow for searching genotype-phenotype correlation. Efforts are in progress in order to perform this analysis. Our data are preliminary and have to be confirmed on a larger population of CF subjects.

We show that AZM treatment does not affect reporter gene activity in either of the tested alleles. Therefore, at least under the experimental conditions we utilized in our assays, the sequence responsible for variable levels of MRP1 detected in other studies [5,8] does not appear to be included in the DNA sequence we analyzed. Previous studies reported that multiple Sp-1 binding domains are located close to *MRP1 *transcriptional start sites near the GCC repeat and might participate in the modulation of transcriptional activity. Zhu and Center [9] suggested a variable length of the GCC repeat in the *MRP1 *5' regulatory region and proposed the influence of this sequence on the *MRP1 *mRNA transcription. In support of this hypothesis, it has been reported that a triple GCC repeat within the 5' flanking region contributes to the regulation of interleukin 1 alpha expression [24]. As Slapak and colleagues demonstrated that *MRP1 *gene amplification is not sufficient for explaining drug resistance [25], the polymorphism we identified might be utilized for investigating whether the number of GCC triplets could be associated with this feature. However, we still cannot exclude a functional relevance of this polymorphism on *MRP1 *mRNA transcription, stability and/or translational efficiency in its native context.

## Conclusion

In summary we established that: 1) the GCC repeat in the *MRP1 *5'UTR is polymorphic, 2) the GCC triplets length do not affect *in vitro *the activity of the promoter in CF airways epithelial cells either in basal conditions or upon AZM treatment.

We propose our experimental for testing whether other molecules potentially relevant for therapy of CF have the capability to influence *MRP1 *gene expression acting on this promoter region.

Finally, the polymorphic length of the GCC repeat we described can be exploited as a genetic marker, possibly linked to additional sequences involved in regulation of constitutive or induced MRP1 expression.

## List of abbreviations

AZM azithromycin

CF cystic fibrosis

CFTR cystic fibrosis transmembrane conductance regulator

GST glutathione S transferase

MRP multidrug resistance-associated protein

UTR untranslated region

## Competing interests

The author(s) declare that they have no competing interests.

## Authors' contributions

EN designed and provided the genotyping method and contributed to the redaction of the manuscript. MP performed the gene reporter analysis and contributed to genotyping. CG participated in the molecular genetic studies. UP collected the clinical data and provided clinical material. BA was involved in supervision of the work. PM designed, coordinated the study and drafted the manuscript.

All authors read and approved the final manuscript.

## Pre-publication history

The pre-publication history for this paper can be accessed here:


